# Obesity‐ and Glucose‐Dependent Differential Autophagy Marker Expression in Adipose Tissues and Adipocytes

**DOI:** 10.1002/osp4.70150

**Published:** 2026-05-23

**Authors:** Edita Islami, Andreas Schmid, Daniel Steger, Sebastian Köhler, Andreas Schäffler, Thomas Karrasch

**Affiliations:** ^1^ Department of Internal Medicine III Giessen University Hospital Giessen Germany

**Keywords:** adipocyte, autophagy, metaflammation, metformin, obesity

## Abstract

**Background:**

Autophagy is an evolutionarily highly conserved process and plays an important role in cellular homeostasis and metabolism. In obesity and obesity‐associated insulin resistance, both enhanced and suppressed autophagy have been observed in adipose tissues (AT).

**Objective and Methods:**

We report on a panel of 7 autophagy markers critically involved in mammalian cellular autophagy in a cohort of patients with obesity (*n* = 40; mean BMI 54.8 ± 6.7 kg/m^2^) versus lean individuals (*n* = 10; mean BMI = 22.7 ± 1.7 kg/m^2^), during 3T3‐L1 adipocyte differentiation in vitro and in mature 3T3‐L1 adipocytes in response to insulin stimulation under physiologic versus high‐glucose conditions in vitro.

**Results:**

Autophagy markers exhibited a differential expression pattern in subcutaneous AT in patients with obesity as compared to patients with normal weight: LAMP2, ATG5, MAP1LC3B and SIRT1 were reduced in patients with obesity, while LAMP1 and SIRT6 were increased. SIRT1 was reduced in visceral versus subcutaneous AT and in patients with obesity with type 2 diabetes mellitus. ATG5 expression in subcutaneous AT correlated positively with systemic HDL cholesterol levels, and LAMP2 and MAP1LC3B expression in subcutaneous AT correlated positively with systemic pro‐inflammatory Meteorin‐like protein levels (METRNL). LAMP1, LAMP2, BECLIN1, ATG5 and MAP1LC3B increased during 3T3 adipocyte differentiation in vitro, while SIRT1 expression decreased. LAMP2 and MAP3LC3B increased in response to insulin stimulation in mature adipocytes under physiological glucose conditions, while LAMP2 expression increased under high‐glucose conditions. Insulin stimulation reduced SIRT1 expression in mature adipocytes under high glucose conditions. Metformin treatment increased BECLIN1 and ATG5 expression in mature adipocytes.

**Conclusions:**

These observations indicate that adipocyte differentiation, glucose and insulin stimulation, pharmacological interventions and local and systemic inflammation associated with obesity differentially impact on individual autophagy markers both on a cellular and systemic level, potentially explaining the inconsistent effects in patients with obesity reported in the literature.

## 1 Introduction

Autophagy is an evolutionary highly conserved process critically involved in cellular homeostasis and metabolism, and changes in autophagy have been observed in multiple inflammatory diseases of both infectious and non‐infectious origin [1, 2, 3]. Generally, autophagy can be divided into microphagy (of cytosolic components directly in lysosomes), chaperone‐mediated autophagy of selective proteins and macrophagy (of multiple cytoplasmic components in double‐membraned autophagosomes). Additionally, selective autophagy of for example mitochondria (mitophagy), endoplasmatic reticulum (ERphagy) or lipids (lipophagy) has been described [4, 5]. In obesity, both activation and suppression of autophagy have been described, hampering a clear identification of its precise role in the pathophysiological processes [1].

Autophagy is involved in both adipocyte development and differentiation and in adipocyte function [5, 6, 7, 8]. The autophagic machinery consists of various separate factors; in the context of obesity, especially ATG5 has been investigated [1, 5, 9, 10, 11]. BECLIN1 and SIRT1 increase autophagy via deacetylation of BECLIN1 and have been implicated in high‐fat diet induced cardiac dysfunction [12, 13]. Since cellular stress and fibrosis are hallmarks of obesity‐associated metabolic inflammation in visceral adipose tissue, MAP1LC3B was investigated for its role in inducing protective autophagy during cellular stress [14], while SIRT6 was selected for protecting from fibroinflammation in obesity via adipocyte browning and lipolysis [15, 16]. LAMP1 was analyzed since it is a putative receptor for the adipokine CTRP3 that mediates anti‐inflammatory effects in adipocytes [17, 18]. Finally, LAMP2 was chosen since its overexpression protects mice from diet‐induced adipose tissue inflammation and insulin resistance obesity, presumably via promoting microlipophagy [19].

Lysosomal membrane associated protein 1 (LAMP1) and 2 (LAMP2) are important components in late stages of the autophagic process, where LAMP1 is localized to late endosomes and lysosomes, and LAMP2 is required for the fusion of autophagosome and lysosome [20]. BECLIN1 is a mammalian autophagy gene (an ortholog of the ATG6 protein in yeast) and plays a central role in autophagy by regulating the formation of a core complex inducing autophagy via its interaction with multiple cofactors [3, 21]. ATG5 is a central autophagy factor indispensable for autophagosome formation and maturation via the ATG5‐ATG12‐ATG16 complex, thereby positively regulating autophagy [22, 23]. Microtubule Associated Protein 1 Light Chain 3 Beta (MAP1LC3B) is located on the phagophore membrane and crucially interacts with autophagy receptors, leading to the formation of the autophagosome membrane. Phosphorylation of LC3B is a key event leading to autophagosome‐lysosome fusion [24]. Sirtuin 1 (SIRT1) is a nicotinamide adenine dinucleotide (NAD+)‐dependent histone deacetylase involved in the regulation of a variety of cellular processes, including cell proliferation, differentiation, autophagy, and cell survival. SIRT1 is particularly closely related to autophagy and increases autophagy via deacetylation of BECLIN1 [13]. Sirtuin 6 (SIRT6) positively regulates autophagy in a variety of cells via modulation of amongst others the AMPK and IGF‐Akt‐mTOR signaling pathways [25, 26].

## 2 Material and Methods

### 2.1 3T3‐L1 Adipocyte Cell Culture

**FIGURE 1:**
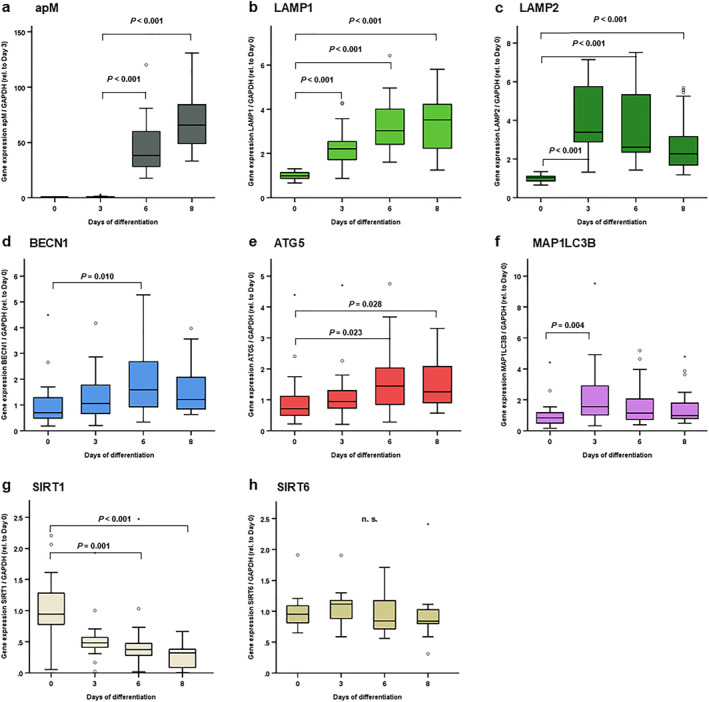
LAMP1, LAMP2, BECN1, ATG5 and MAP1LC3B expression levels were induced during 3T3‐L1 adipocyte differentiation in vitro, whereas SIRT1 expression was reduced. 3T3‐L1 pre‐adipocytes were cultured and differentiated into mature adipocytes as described in the Methods section. At the indicated time points (0 = pre‐adipocytes, d3/d6 = during the differentiation process, d8 = mature adipocytes), total RNA was isolated and mRNA expression levels of autophagy markers were assessed using reverse transcription of RNA and real‐time PCR (RT‐PCR) and were normalized to GAPDH expression in all samples. Gene expression data were analyzed using *Kruskal–Wallis* test (for comparison of > 2 independent samples) and Bonferroni correction for multiple testing was applied (adjusted *p* values are shown). (a) Increased Adiponectin (apM) expression during the differentiation process is shown as a control. LAMP1 (b), LAMP2 (c), BECN1 (d), ATG5 (e) and MAP1LC3B (f) were increased at different time points during the adipocyte differentiation process, as indicated. (g) SIRT1 was reduced during the adipocyte differentiation. (H) No differences were observed in SIRT6 expression levels during the differentiation process. N = 16–24; *p* values are indicated in the respective figure panels.

3T3‐L1 pre‐adipocytes [27] were cultured and differentiated into mature adipocytes as described previously [28]. Briefly, cells were cultured at 37°C and 5% CO_2_ in Dulbecco's Modified Eagle Medium (DMEM, Biochrom AG, Berlin, Germany) supplemented with 10% newborn calf serum (NCS, Sigma‐Aldrich, Deisenhofen, Germany) and 1% penicillin/streptomycin (Aidenbach, Germany) and were differentiated into adipocytes in DMEM/F12/glutamate medium (Lonza, Basel, Switzerland) supplemented with 20 μM 3‐isobutyl‐methyl‐xanthine (Serva, Heidelberg, Germany), 1 μM corticosterone, 100 nM insulin, 200 μM ascorbate, 2 μg/mL transferrin, 5% fetal calf serum (FCS, Sigma‐Aldrich, Deisenhofen, Germany), 1 μM biotin, 17 μM pantothenate, 100 nM rosiglitazone (all from Sigma Aldrich, Deisenhofen Germany), and 300 μg/mL Pedersen‐fetuin (MP Biomedicals, Illkirch, France) [29, 30]. A differentiation protocol reported in the literature [27, 31, 32, 33, 34] was used with slight modifications over a duration of 8 days. Cell phenotype was controlled via light‐microscopy throughout the process of differentiation. Analyses during 3T3‐L1 adipocyte differentiation (Figure 1) were performed in *n* = 24 independent samples per condition/differentiation day.

**FIGURE 2:**
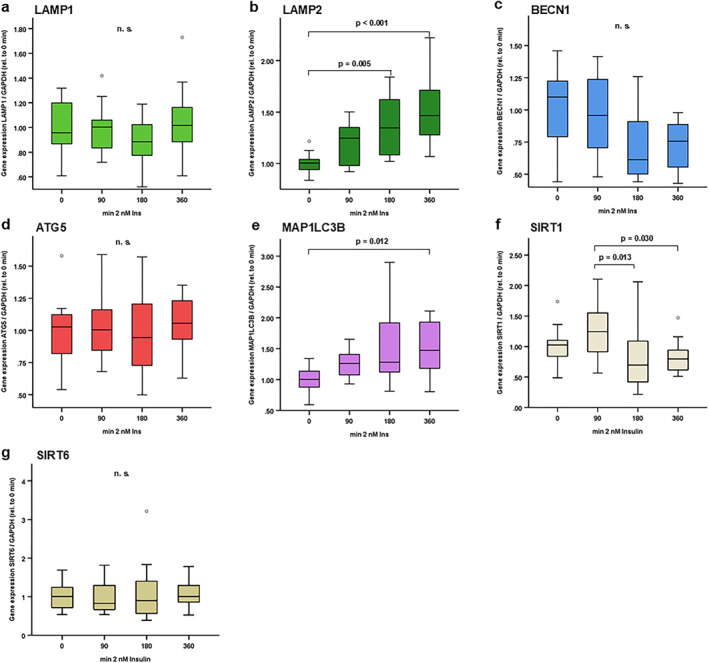
LAMP2 and MAP1LC3B autophagy markers were induced in response to insulin treatment in adipocytes under physiological glucose conditions (1.0 g/L). 3T3‐L1 pre‐adipocytes were cultured and differentiated into mature adipocytes as described in the Methods section. Mature adipocytes were incubated in serum‐free DMEM/F12 medium for 24 h prior to stimulation experiments, and cells were then treated with insulin (2 nM) for different incubation periods (from 90 to 360 min) in serum‐free medium containing regular glucose levels (1.0 g/L, 100 mg/dL, 5.6 mmol/L). At the indicated time points, total RNA was isolated and mRNA expression levels of autophagy markers were assessed using reverse transcription of RNA and real‐time PCR (RT‐PCR) and were normalized to GAPDH expression in all samples. Gene expression data were analyzed using *Kruskal–Wallis* test (for comparison of > 2 independent samples) and Bonferroni correction for multiple testing was applied (adjusted *p* values are shown). LAMP2 (b) and MAP1LC3B (e) were induced at different time points after insulin treatment. SIRT1 (f) was reduced after 180 and 360 min as compared to the 90 min tome point. No significant impact of insulin treatment was observed on LAMP1 (a), BECN1 (c), ATG5 (d) and SIRT6 (g) under these conditions. N = 12–16; *p* values are indicated in the respective figure panels.

**FIGURE 3:**
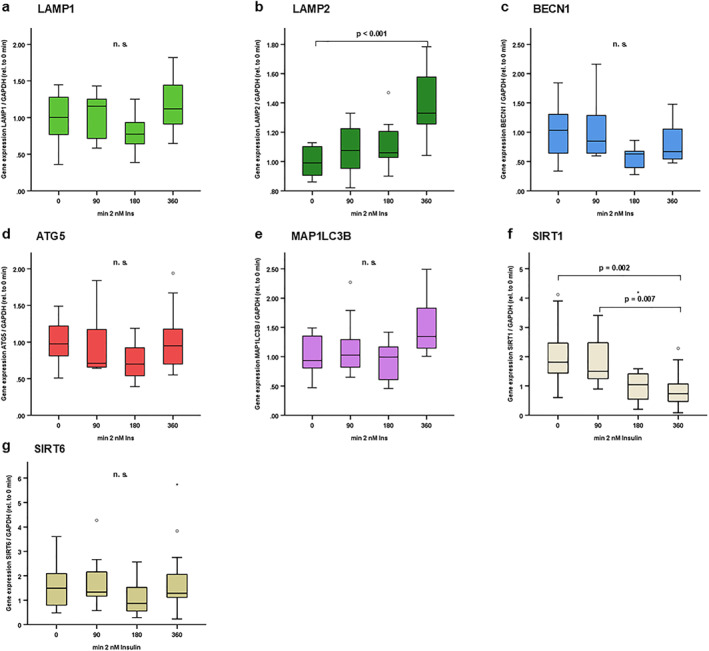
LAMP2 was induced while SIRT1 was reduced in response to insulin treatment in adipocytes under high glucose conditions (4.5 g/L). 3T3‐L1 pre‐adipocytes were cultured and differentiated into mature adipocytes as described in the Methods section. Mature adipocytes were incubated in serum‐free DMEM/F12 medium for 24 h prior to stimulation experiments, and cells were then treated with insulin (2 nM) for different incubation periods (from 90 to 360 min) in serum‐free medium containing high glucose levels (4.5 g/L, 450 mg/dL, 25 mmol/L). At the indicated time points, total RNA was isolated and mRNA expression levels of autophagy markers were assessed using reverse transcription of RNA and real‐time PCR (RT‐PCR) and were normalized to GAPDH expression in all samples. Gene expression data were analyzed using *Kruskal–Wallis* test (for comparison of > 2 independent samples) and Bonferroni correction for multiple testing was applied (adjusted *p* values are shown). LAMP2 (b) was induced 360 min after insulin treatment. SIRT1 (f) was reduced 360 min after insulin treatment. No significant impact of insulin treatment was observed on LAMP1 (a), BECN1 (c), ATG5 (d), MAP1LC3B (e) and SIRT6 (g) under these conditions. N = 12–17; *p* values are indicated in the respective figure panels.

**FIGURE 4:**
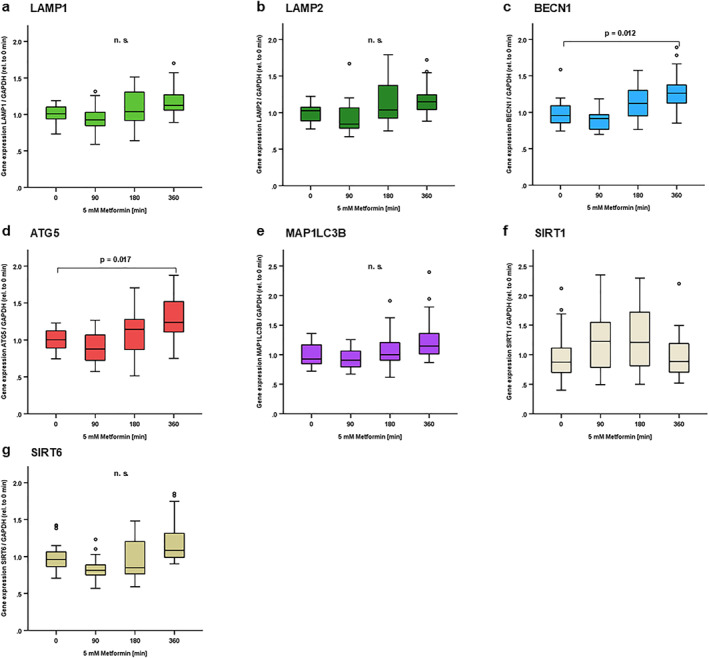
BECLIN1 and ATG5 autophagy markers were induced in response to metformin treatment in adipocytes. 3T3‐L1 pre‐adipocytes were cultured and differentiated into mature adipocytes as described in the Methods section. Mature adipocytes were incubated in serum‐free DMEM/F12 medium for 24 h prior to stimulation experiments, and cells were then treated with metformin (5 mM) for different incubation periods (from 90 to 360 min) in serum‐free medium containing intermediate high glucose levels (3.1 g/L, 314.1 mg/dL, 17.4 mmol/L). At the indicated time points, total RNA was isolated and mRNA expression levels of autophagy markers were assessed using reverse transcription of RNA and real‐time PCR (RT‐PCR) and were normalized to GAPDH expression in all samples. Gene expression data were analyzed using *Kruskal–Wallis* test (for comparison of > 2 independent samples) and Bonferroni correction for multiple testing was applied (adjusted *p* values are shown). BECLIN1 (c) and ATG5 (d) were induced at 360 min after metformin treatment. No significant impact of metformin treatment was observed on LAMP1 (a), LAMP2 (b), MAP1LC3B (e), SIRT1 (f) and SIRT6 (g) under these conditions. N = 12; *p* values are indicated in the respective figure panels.

Mature adipocytes were incubated in serum‐free DMEM/F12 medium for 24 h prior to stimulation experiments. Cells were then treated with insulin (2 nM) for different incubation periods (from 90 to 360 min) and with different doses of glucose in serum‐free medium (1.0 and 4.5 g/L), as specified in the respective figure legends. In a different set of experiments, mature adipocytes were incubated with 5 mM Metformin (from Sigma Aldrich, Deisenhofen, Germany) in serum‐free medium containing 3.1 g/L glucose for different incubation periods (from 90 to 360 min). Stimulation experiments in mature 3T3‐L1 adipocytes (Figures 2, 3, 4) were performed in at least *n* = 12 independent samples per condition.

To exclude unexpected effects on cell viability, LDH (lactate dehydrogenase) activity was measured in cell supernatants (Cytotoxicity Detection Kit, Roche, Mannheim, Germany).

### 2.2 Clinical Cohort

Anthropometric and clinical data as well as serum samples, subcutaneous adipose tissue (scAT) and visceral adipose tissue (visAT) specimens were retrieved from *n* = 40 patients with obesity without an acute illness (mean BMI 54.8 ± 6.7 kg/m^2^, 8 male/32 female, type 2 diabetes mellitus (T2D) in 17 patients) undergoing metabolic surgical therapy from the previously described *Research in Obesity and Bariatric Surgery* (ROBS) patient cohort [35] and applied for correlation and subgroup analysis. The study was approved by the local ethical committee at the University of Giessen, Germany (AZ 101/14). All methods were performed in accordance with the relevant guidelines and regulations, and studies were performed in accordance with the Declaration of Helsinki. All patients gave informed consent and were informed about the aim of the study. Data anonymization and privacy policy were accurately applied.

As a comparison group, subcutaneous adipose tissue (scAT) mRNA samples from *n* = 10 patients without an acute illness and with normal‐weight (BMI = 22.7 ± 1.7 kg/m^2^, 2 male/8 female, T2D in 0 patients) were purchased from BioCat (Heidelberg, Germany) and included in our analyses. Comparison patients were similar in age and sex distribution. Supporting Information S1: Table S1 gives a brief overview over patient demographics in the respective groups.

### 2.3 RNA Isolation and Real‐Time RT‐PCR

Total RNA was isolated from mature adipocytes and from different adipose tissues as was reported previously [28]. Briefly, cells and/or tissues were homogenized in TRIzol‐Reagent (Life Technologies GmbH, Darmstadt, Germany) in combination with gentleMACS dissociator and M‐tubes (Miltenyi Biotec GmbH, Bergisch Gladbach, Germany) for dissociation and RNA was isolated applying RNeasy Mini Kit (Qiagen, Hilden, Germany) including DNase digestion (RNase‐Free DNase Set, Qiagen, Hilden, Germany). For gene expression analysis, reverse transcription of RNA (QuantiTect Reverse Transcription Kit from Qiagen, Hilden, Germany) was performed to generate corresponding cDNA for real‐time PCR (RT‐PCR) (iTaq Universal SYBR Green Supermix, CFX Connect RT‐PCR system; Bio‐Rad, Munich, Germany).

Target gene mRNA levels in 3T3‐L1 adipocytes were quantified using real‐time RT‐PCR and normalized to expression of the house‐keeping gene Glyceraldehyde 3‐phosphate dehydrogenase (GAPDH) applying ddCT method. Each sample was analyzed in triplicates and results with an intra‐triplicate standard deviation exceeding 0.5 quantification cycles were excluded from further analysis. All oligonucleotides used were purchased from Metabion, Martinsried, Germany. Primer sequences are provided in Supporting Information S1: Table S2.

### 2.4 Adipokine and Cytokine ELISA Measurements

Human Adiponectin, C1q/TNF‐related protein 3 (CTRP3), LL‐37 (the active form of Cathelicidin antimicrobial peptide (CAMP)), Meteorin‐like protein (METRNL), and Resistin serum levels were assessed in serum samples of *n* = 40 obese patients before metabolic surgery via ELISA techniques (DuoSet Development Kits from Biotechne, Wiesbaden, Germany; LL37 ELISA Kit from Hycultec, Beutelsbach, Germany). Human C‐reactive protein (CRP), total cholesterol, LDL cholesterol, HDL cholesterol and triglycerides as well as HbA_1c_ levels were assessed in serum samples of *n* = 40 obese patients before metabolic surgery were measured at the Institute of Clinical Chemistry and Laboratory Medicine, University of Giessen, Germany, as reported previously [35].

### 2.5 Statistical Analysis

The software package *SPSS 29.0* was applied for statistical data analysis. Gene expression data were analyzed by via *Mann‐Whitney* test and *Kruskal–Wallis* test (for comparison of 2 and > 2 independent samples, respectively), *Wilcoxon* test (for comparison of 2 paired samples) and *Pearson's chi‐squared* test, and Bonferroni's correction was applied. Correlation analysis was performed by Spearman‐rho test, and *p* values were corrected for multiple testing using Bonferroni correction. In the figures, data are given as box‐plots indicating median and interquartile ranges. Linear variables were analyzed by *Spearman‐rho* rank correlation test and correlation data are presented as dot plots. Corrected *p* values below 0.05 were considered statistically significant.

## 3 Results

### 3.1 LAMP1 and SIRT6 Were Induced in scAT in Patients With Obesity

**FIGURE 5:**
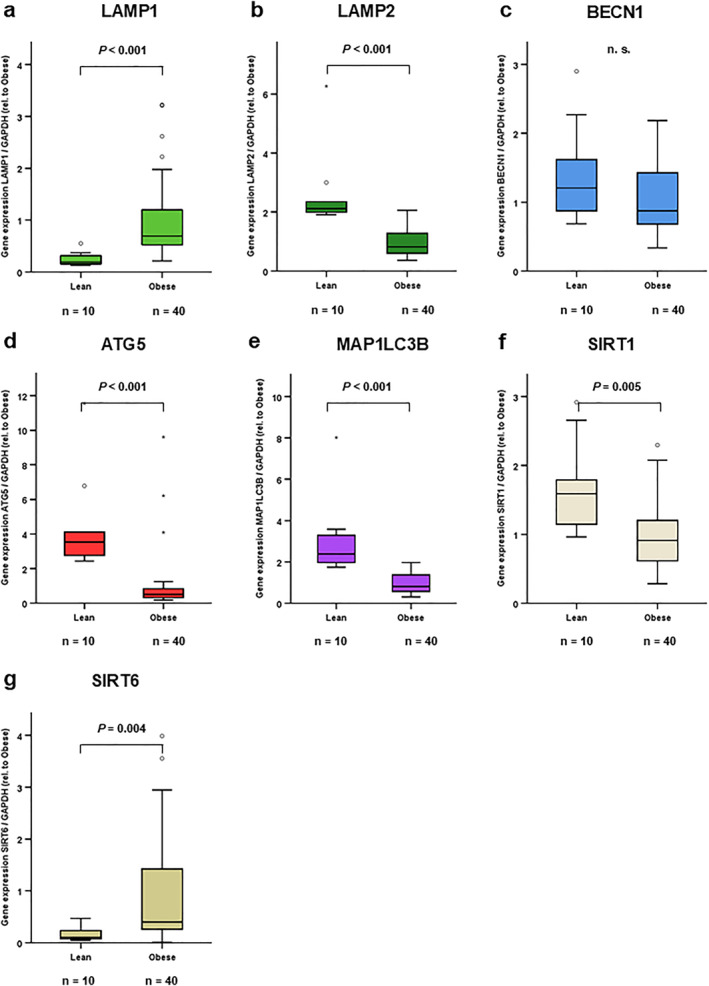
LAMP1 and SIRT6 autophagy gene expression were induced while LAMP2, ATG5, MAP1LC3B and SIRT1 expression were reduced in subcutaneous adipose tissue of patients with obesity compared to lean controls. mRNA was extracted from total subcutaneous adipose tissue (scAT) in patients with severe obesity (*n* = 40; mean BMI 54.8 ± 6.7 kg/m^2^). For comparison, mRNA extracted from total subcutaneous adipose tissue (scAT) in lean individuals (*n* = 10; mean BMI 22.7 ± 1.7 kg/m^2^) was purchased from BioCat (Heidelberg, Germany). mRNA expression levels of autophagy markers were assessed using reverse transcription of RNA and real‐time PCR (RT‐PCR) and were normalized to GAPDH expression in all samples. Gene expression data were analyzed using *Mann‐Whitney* test (for comparison of 2 independent samples) and Bonferroni correction for multiple testing was applied (adjusted *p* values are shown). Supporting Information S1: Table S3 displays expression levels relative to GAPDH in both groups; for graphical demonstration in this figure, expression levels were normalized to the expression levels in patients with obesity. LAMP1 (a) and SIRT6 (g) were increased in patients with obesity versus normal weight controls, while LAMP2 (b), BECN1 (c), ATG5 (d), MAP1LC3B (e) and SIRT1 (f) were reduced. *p* values are indicated in the respective figure panels.

Relative mRNA concentrations of LAMP1, LAMP2, BECN1, ATG5, MAP1LC3B, SIRT1 and SIRT6 were compared in total subcutaneous adipose tissue (scAT) between patients with normal weight (*n* = 10; mean BMI = 22.7 ± 1.7 kg/m^2^) and patients with obesity (*n* = 40; mean BMI 54.8 ± 6.7 kg/m^2^). As is illustrated in Figure 5, expression of autophagy‐associated LAMP1 and SIRT6 was induced in scAT of patients with obesity (Figure 5a,g). Expression levels of the autophagy marker genes LAMP2, ATG5, MAP1LC3B and SIRT1 were significantly reduced (Figure 5b,d–f), while BECN1 remained unaffected (Figure 5c).

### 3.2 SIRT1 Was Reduced in visAT in Patients With Obesity

**FIGURE 6:**
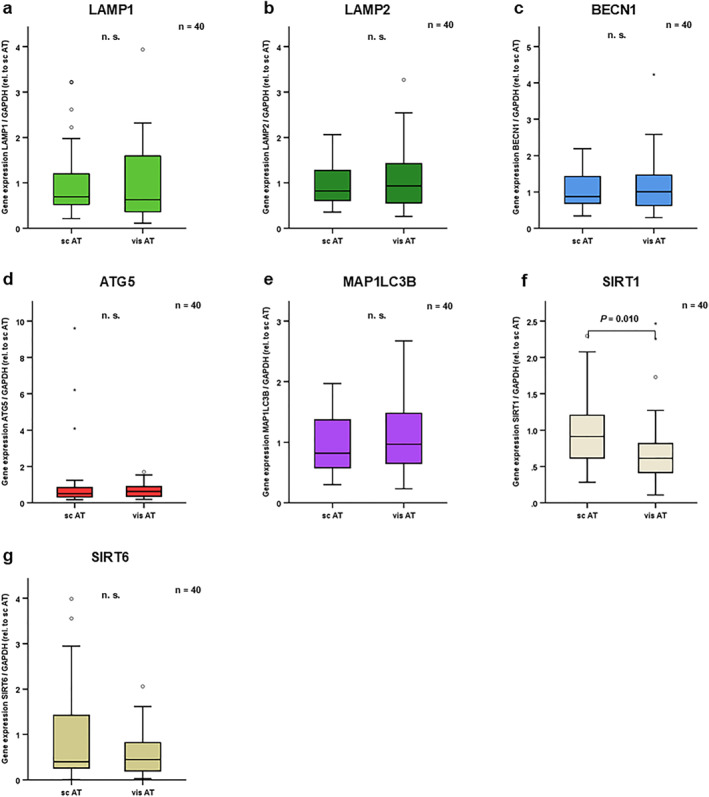
SIRT1 expression levels were reduced in visceral adipose tissue as compared to subcutaneous adipose tissue in patients with obesity. mRNA was extracted from total subcutaneous adipose tissue (scAT) as well as total visceral adipose tissue (visAT) in patients with severe obesity (*n* = 40; mean BMI 54.8 ± 6.7 kg/m^2^). mRNA expression levels of autophagy markers were assessed using reverse transcription of RNA and real‐time PCR (RT‐PCR) and were normalized to GAPDH expression in all samples. Gene expression data were analyzed using *Wilcoxon* test and Bonferroni correction for multiple testing was applied (adjusted *p* values are shown). Supporting Information S1: Table S4 displays expression levels relative to GAPDH in both groups; for graphical demonstration in this figure, expression levels were normalized to expression in scAT. SIRT1 expression levels were reduced (f) in visAT as compared to scAT. No differences between scAT and visAT were observed in LAMP1 (a), LAMP2 (b), BECN1 (c), ATG5 (d), MAP1LC3B (e) and SIRT6 (g) expression levels. *p* values are indicated in the respective figure panels.

Subsequent comparative analyses focused on autophagy marker expression in different adipose tissues in patients with obesity (*n* = 40; mean BMI 54.8 ± 6.7 kg/m^2^). Expression of most autophagy marker genes was similar in scAT and visceral adipose tissue (visAT) in obesity (Figure 6a–e,g). However, SIRT1 expression was significantly reduced in visAT as compared to scAT (Figure 6f).

LAMP1, LAMP2, BECN1, ATG5 and MAP1LC3B gene expression levels strongly correlated with each other within the subcutaneous and the visceral adipose tissues, respectively (Table 1). However, autophagy regulator SIRT1 and SIRT6 gene expression did not correlate to the other autophagy markers in either AT depot (data not shown).

**TABLE 1:** Correlations between mRNA levels of autophagy marker genes in subcutaneous (scAT) and visceral adipose tissue (visAT) of patients with obesity.

	LAMP1		LAMP2		BECN1		ATG5		MAP1LC3B	
visAT mRNA	*rho*	*p*	*rho*	*p*	*rho*	*p*	*rho*	*p*	*rho*	*p*
LAMP1			+ 0.702	< 0.001	+ 0.720	< 0.001	+ 0.612	< 0.001	+ 0.450	0.025
LAMP2	+ 0.702	< 0.001			+ 0.622	< 0.001	+ 0.814	< 0.001	+ 0.864	< 0.001
BECN1	+ 0.720	< 0.001	+ 0.622	< 0.001			+ 0.677	< 0.001	+ 0.426	0.043
ATG5	+ 0.612	< 0.001	+ 0.814	< 0.001	+ 0.677	< 0.001			+ 0.697	< 0.001
MAP1LC3B	+ 0.450	0.025	+ 0.864	< 0.001	+ 0.426	0.043	+ 0.697	< 0.001		
scAT mRNA										
LAMP1			+ 0.610	< 0.001	+ 0.495	0.008	+ 0.549	0.002	+ 0.478	0.013
LAMP2	+ 0.610	< 0.001			+ 0.819	< 0.001	+ 0.873	< 0.001	+ 0.884	< 0.001
BECN1	+ 0.495	0.008	+ 0.819	< 0.001			+ 0.830	< 0.001	+ 0.771	< 0.001
ATG5	+ 0.549	0.002	+ 0.873	< 0.001	+ 0.830	< 0.001			+ 0.858	< 0.001
MAP1LC3B	+ 0.478	0.013	+ 0.884	< 0.001	+ 0.771	< 0.001	+ 0.858	< 0.001		

*Note:* Non‐parametric Spearman‐*rho* test was applied for calculation of correlation coefficients and statistical significance, and *p* values have been corrected for multiple testing using Bonferroni correction (*n* = 40; mean BMI 54.8 ± 6.7 kg/m^2^).

### 3.3 SIRT1 Gene Expression in scAT Was Reduced in Patients With T2D

**FIGURE 7:**
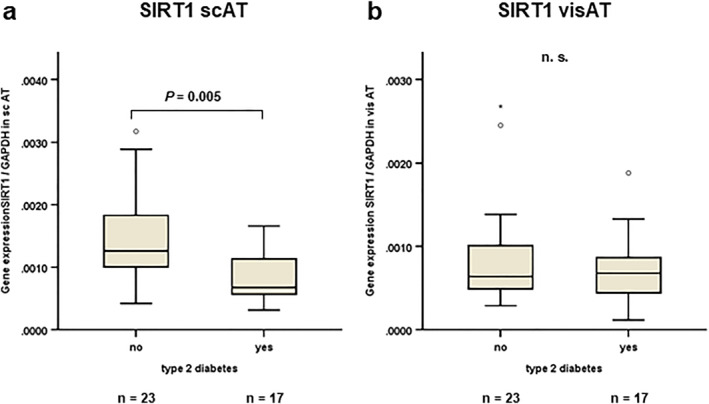
SIRT1 gene expression in subcutaneous adipose tissue was reduced in patients with obesity with T2D. mRNA was extracted from total subcutaneous adipose tissue (scAT) as well as total visceral adipose tissue (visAT) in patients with severe obesity (*n* = 40; mean BMI 54.8 ± 6.7 kg/m^2^). mRNA expression levels of SIRT1 were assessed using reverse transcription of RNA and real‐time PCR (RT‐PCR) and were normalized to GAPDH expression. Gene expression data were analyzed using *Mann–Whitney* test and Bonferroni correction for multiple testing was applied (adjusted *p* values are shown). (a) SIRT1 expression was reduced in patients with T2D (*n* = 17) as compared to patients without T2D (*n* = 23) (*p* = 0.005). (b) Reduced SIRT1 expression in patients with T2D was not observed in visAT (*p* = not significant).

Subgroup analysis did not reveal a significant impact of gender (*n* = 8 male/32 female) on expression levels of autophagy markers in scAT and visAT (data not shown). However, SIRT1 mRNA concentration in scAT was significantly reduced in patients with T2D (*n* = 17 of 40 patients) (*p* = 0.005) (Figure 7a). Reduced SIRT1 expression was not observed in visAT (Figure 7b).

### 3.4 ScAT Autophagy Markers Correlated With Systemic HDL Cholesterol and METRNL Levels

Autophagy interacts with critical pathophysiological processes during adipose tissue pro‐inflammatory changes in obesity and systemic insulin resistance [1, 5, 11, 36, 37]. Thus, further analyses asked if autophagy marker gene expression correlated with anthropometric and clinical parameters (age, body weight, waist‐to‐hip ratio, BMI and serum lipids total cholesterol, triglycerides, low‐density lipoprotein cholesterol (LDL) and high‐density lipoprotein cholesterol (HDL)) as well as separately with cytokine markers of adipose inflammation in the systemic circulation in patients with obesity (Adiponectin, Resistin, C‐reactive protein (CRP); LL‐37 (the active form of Cathelicidin antimicrobial peptide (CAMP)), C1q/TNF‐related protein 3 (CTRP3), Meteorin‐like protein (METRNL)).

**FIGURE 8:**
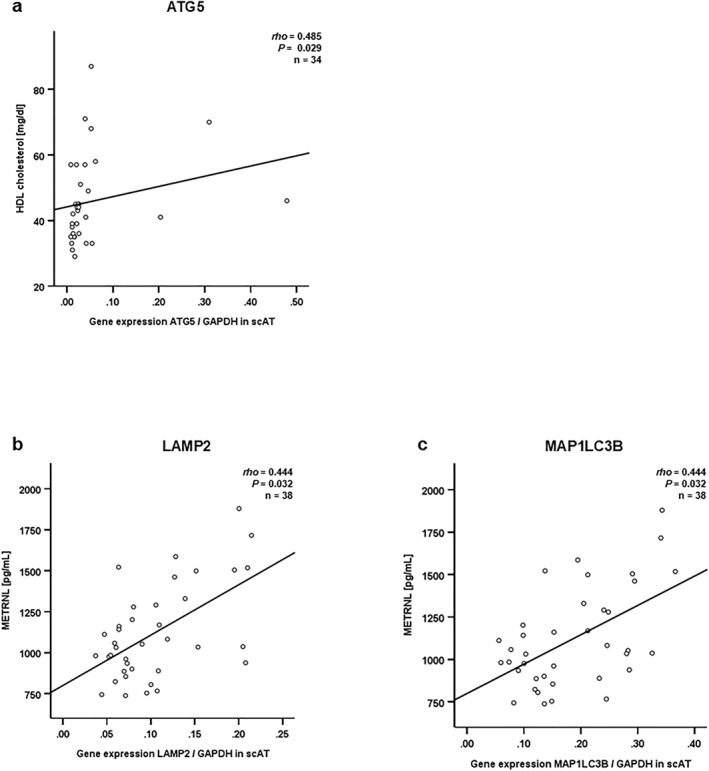
ATG5 in subcutaneous adipose tissue was positively correlated to systemic HDL cholesterol levels, while LAMP2 and MAP1LC3B were positively correlated to systemic METRNL levels. mRNA was extracted from total subcutaneous adipose tissue (scAT) in patients with severe obesity. mRNA expression levels of autophagy markers were assessed using reverse transcription of RNA and real‐time PCR (RT‐PCR) and were normalized to GAPDH expression in all samples. Non‐parametric Spearman‐rho test was applied for calculation of correlation coefficients and statistical significance for anthropometric and clinical parameters (age, body weight, waist‐to‐hip ratio, BMI and serum lipids total cholesterol, triglycerides, low‐density lipoprotein cholesterol (LDL) and high‐density lipoprotein cholesterol (HDL)), and *p* values have been corrected for multiple testing using Bonferroni correction (adjusted *p* values are shown). In a separate analysis, non‐parametric Spearman‐rho test was applied for calculation of correlation coefficients and statistical significance for cytokine markers of adipose inflammation in the systemic circulation (Adiponectin, Resistin, C‐reactive protein (CRP); LL‐37 (the active form of Cathelicidin antimicrobial peptide (CAMP)), C1q/TNF‐related protein 3 (CTRP3), Meteorin‐like protein (METRNL)), and *p* values have been corrected for multiple testing using Bonferroni correction (adjusted *p* values are shown). (a) ATG5 expression was positively correlated to systemic HDL cholesterol levels (rho = 0.485, *p* = 0.029). (b) LAMP2 and (c) MAP1LC3B expression were positively correlated to systemic METRNL levels (rho = 0.444, *p* = 0.032 and rho = 0.444, *p* = 0.032, respectively).

No significant correlations of AT autophagy markers with the anthropometric and metabolic parameters age, body weight, waist‐to‐hip ratio, BMI and serum lipids apart from systemic high‐density lipoprotein cholesterol (HDL) were observed (data not shown). Similarly, Adiponectin, Resistin, CRP, LL‐37, CTRP3 did not correlate to AT autophagy markers (data not shown). However, ATG5 expression levels in scAT exhibited a significant positive correlation with systemic HDL levels (*p* = 0.029 after correction for multiple testing) (Figure 8a). LAMP2 and MAP1LC3B in scAT positively correlated with systemic METRNL levels (Figure 8b,c).

### 3.5 Differential Autophagy Marker Regulation During Adipocyte Differentiation In Vitro

Autophagy is implicated both in pro‐inflammatory changes in adipose tissues in obesity as well as in adipocyte differentiation and physiology [1, 7, 8, 36, 37, 38]. Considering the significant regulation of autophagy markers in adipose tissues in vivo, further studies focused on the expression of these autophagy markers in adipocytes in vitro. LAMP1, LAMP2, BECN1, ATG5, MAP1LC3B, SIRT1 and SIRT6 mRNA concentrations were quantified during hormonally induced adipocyte differentiation of 3T3‐L1 cells, while adipocyte differentiation and maturing was reflected by adiponectin gene induction (Figure 1a). LAMP1, LAMP2, BECN1, ATG5 and MAP1LC3B expression levels were upregulated at different time points during adipocyte differentiation: While LAMP1 and LAMP2 were induced early (d3) and persistently (through d8) during differentiation (Figure 1b,c), BECN1 and ATG5 were upregulated at later differentiation stages (d6; Figure 1d,e). MAP1LC3B was only transiently induced at early differentiation stages (d3; Figure 1f).

SIRT1 exhibited different dynamics, with a steady and considerable decline from day 0 to day 8 (Figure 1g). SIRT6 expression levels remained unaffected during adipocyte differentiation (Figure 1h).

### 3.6 Differential Autophagy Marker Regulation in Response to Insulin In Vitro

Since autophagy is implicated in adipocyte physiology [7, 8, 38, 39, 40], additional experiments investigated autophagy marker gene expression in response to insulin stimulation for 90–360 min under conditions of physiological glucose concentration (1.0 g/L, 100 mg/dL, 5.6 mmol/L; Figure 2a–g) versus high glucose concentration (4.5 g/L, 450 mg/dL, 25 mmol/L; Figure 3a–g) in mature 3T3‐L1 adipocytes in vitro. LAMP2 and MAP1LC3B were significantly induced by insulin in a time‐dependent manner—after 6 h—at physiological glucose concentrations (1.0 g/L; Figure 2b,e), whereas SIRT1 was reduced at 3 and 6 h as compared to 1.5 h time points (Figure 2f). LAMP1, BECN1, ATG5 and SIRT6 were not regulated by insulin treatment (Figure 2a,c,d,g). LAMP2 upregulation in response to insulin treatment was also observed in a high glucose setting (4.5 g/L; Figure 3b), while MAP1LC3B was not significantly induced under these conditions (Figure 3e). Interestingly, there was a considerable and time‐dependent decline in SIRT1 mRNA concentrations in response to insulin treatment under high glucose conditions (4.5 g/L; Figure 3f).

High glucose concentration (4.5 g/L, 450 mg/dL, 25 mmol/L) without insulin treatment compared to physiological glucose concentration (1.0 g/L, 100 mg/dL, 5.6 mmol/L) did not significantly impact on basal LAMP1, BECN1, ATG5, MAP1LC3B, SIRT1 and SIRT6 expression after correction for multiple testing (data not shown). However, we observed a weak, albeit significant induction of LAMP2 expression in high glucose as compared to regular glucose concentration (gene expression LAMP2/GAPDH ratio was 0.0184 ± 0.0031 vs. 0.0147 ± 0.0048, *p* = 0.03 after correction for multiple testing).

### 3.7 Metformin Induced BECLIN1 and ATG5 in Adipocytes

Metformin is a pharmacologic agent commonly used in patients with T2D and insulin resistance, where it sensitizes adipocytes to insulin signaling [41]. Interestingly, studies have shown that metformin both induced and inhibited autophagy in various other cell types [42]. Therefore, autophagy marker expression was analyzed in response to metformin stimulation for 90–360 min under conditions of intermediate high glucose concentration (3.1 g/L, 314 mg/dL, 17.4 mmol/L) in mature 3T3‐L1 adipocytes in vitro. BECLIN1 and ATG5 were significantly induced by metformin in a time‐dependent manner—after 6 h—(Figure 4c,d), whereas LAMP1, LAMP2, MAP1LC3B, SIRT1 and SIRT6 were not regulated by metformin treatment (Figure 4a,b,e–g).

## 4 Discussion

The current study provides a targeted expression analysis of 7 different autophagy markers, which were selected for being critically involved in mammalian cellular autophagy and in metabolically induced adipose tissue inflammation at different stages: LAMP1, LAMP2, BECLIN1, ATG5, MAP1LC3B, SIRT1 and SIRT6. Analyses were performed in subcutaneous versus visceral adipose tissues of patients with obesity (*n* = 40; mean BMI 54.8 ± 6.7 kg/m^2^) versus patients with normal body weight (*n* = 10; mean BMI = 22.7 ± 1.7 kg/m^2^), and in vitro during 3T3‐L1 adipocyte differentiation. In additional experiments, autophagy marker expression was investigated in 3T3‐L1 adipocytes under *high‐glucose* versus *physiological‐glucose* conditions ± insulin stimulation and after metformin treatment. A differential expression pattern of autophagy markers was observed in obesity and during adipocyte differentiation. Glucose concentration modulated adipocyte autophagy marker expression in vitro.

Autophagy markers positively correlated with each other within both subcutaneous and visceral adipose tissues (AT) except for SIRT1 and SIRT6 (Table 1). Autophagy markers LAMP2 and MAP1LC3B in subcutaneous AT correlated positively with pro‐inflammatory METRNL in the systemic circulation (Figure 8b,c), indicating that autophagy in AT correlated to systemic inflammation. These data are in line with earlier studies describing an important role of autophagy in inflammation‐dependent metabolic regulation and lipid metabolism [1, 43, 44] in which autophagy correlated with AT inflammation in obesity [9, 36]. Interestingly, ATG5 positively correlated with systemic HDL cholesterol levels, which are protective in cardiovascular disease (Figure 8a).

Autophagy markers LAMP1 and SIRT6 were increased in subcutaneous AT of patients with obesity as compared to patients with normal body weight (Figure 5). No differences were observed in LAMP1 and SIRT6 expression levels between subcutaneous and visceral AT in patients with obesity (Figure 6). Since LAMP1 localizes to endosomes and lysosomes in the late autophagy process [20], while SIRT6 positively regulates autophagy via AMPK and IGF‐Akt‐mTOR signaling [25, 26], these data are compatible with increased autophagy in AT in obesity. However, since SIRT6 deficiency impairs browning of white adipose tissue and sensitizes mice to high‐fat diet‐induced obesity and insulin resistance [45, 46], increased adipose tissue SIRT6‐expression might also serve protective roles in obesity.

Critical autophagy regulators ATG5 and MAP1LC3B are indispensable for autophagosome formation [22, 23, 24]. Surprisingly, overall ATG5 as well as MAP1LC3B expression was reduced in subcutaneous adipose tissues in patients with obesity versus normal weight (Figure 5d,e). Other groups reported increased ATG5 and LC3B expression in human adipose tissues in obesity [9, 10, 11]. The findings reported here are matched by observations in rat AT after feeding a diet high in fructose for 5 months, where MAP1LC3B was reduced [47]. It has been remarked that the expression levels of autophagy markers do not necessarily reflect autophagic flux as a marker of autophagic activity [48]: Autophagic flux was reported to be reduced in human obesity [49]. Consequently, to date data in the literature are conflicting, owing to the complex interplay between autophagy, local and systemic inflammation and metabolism [1, 44, 50].

LAMP2, BECN1 and SIRT1 expression levels were reduced in subcutaneous AT in patients with obesity as compared to normal weight (Figure 5). Mechanistically, fasting is suggested to induce BECN1 expression via AMPK induction and subsequent Ulk‐1 activation, whereas obesity is associated with reduced BECN1 expression. This downregulation of BECN1 is mediated directly by JNK and indirectly by PI3K/Akt‐related mTORC1 upregulation, which in turn inhibits Ulk‐1 activation in obesity [1]. Aijala and colleagues found reduced LAMP2 expression in rat AT after feeding a diet high in fructose for 5 months [47]. Since LAMP2 is required in the late autophagy process for the fusion of autophagosome and lysosome [20], while BECLIN1 regulates the formation of the autophagy core complex [3, 21], these data hint at potentially reduced autophagy in subcutaneous AT in patients with obesity in the current study. These observations are in line with the observed reduction in SIRT1 expression levels. No differences were observed in LAMP2 and BECLIN1 expression in subcutaneous versus visceral AT in our patients, while SIRT1 was reduced in visceral AT (Figure 6).

Since SIRT1 is involved in cell proliferation, differentiation and survival in addition to autophagy [13], additional homeostatic processes other than autophagy impact on the SIRT1 expression levels. Interestingly, SIRT1 negatively regulates macrophage infiltration into AT as a hallmark of metabolically‐induced AT inflammation in diet‐induced obesity [51, 52] and reduces systemic insulin resistance [53]. In line with these reports, reduced SIRT1 expression was observed in subcutaneous AT in patients with type 2 diabetes mellitus (T2D) (Figure 7a).

LAMP1 expression was increased in scAT in patients with obesity as compared to lean controls, while LAMP2 expression was reduced (Figure 5a,b). No differences were observed in LAMP1 or LAMP2 expression in scAT versus visAT in patients with obesity (Figure 6a,b). LAMP1 and LAMP2 expression strongly correlated to each other within the visceral and separately within the subcutaneous AT in patients with obesity (Table 1). Future studies should include an analysis of autophagy marker expression in visceral and subcutaneous AT specimen of patients with normal weight to elaborate on these observations.

Adipocyte cellular development and differentiation from mesenchymal stem cells into mature adipocytes is a highly complex process closely regulated by differentiation‐phase specific transcription factors. These adipogenic transcription factors in turn modulate autophagy gene expression, which plays an important role during adipocyte differentiation [7, 8, 39, 40]. For example, knock‐down of autophagy genes ATG5 or ATG7 impaired adipocyte differentiation in mice [54, 55]. During 3T3‐L1 preadipocyte differentiation into mature adipocytes in vitro, a significantly increased expression of LAMP1, LAMP2, BECN1, ATG5 and MAP1LC3B was observed at different time points during the differentiation process (Figure 1). LAMP1 and LAMP2 expression were significantly elevated during the entire differentiation, with a maximum expression of LAMP1 on day 8 versus LAMP2 on day 3. On the other hand, BECLIN1 and ATG5 increased late in the differentiation process on day 6 and day 8. MAP1LC3B was found at increased expression levels early in the differentiation process on day 3. SIRT1 expression pattern differed showing generally reduced levels, while SIRT6 expression remained unchanged during adipocyte differentiation.

Apart from cellular adipocyte differentiation, autophagy also plays an important role in adipocyte function [1, 5, 38]: For example, autophagy was shown to be dysregulated in patients with obesity and in obesity‐associated insulin resistance with increased expression levels of autophagy markers ATG5, ATG7 and LC3B [11, 56, 57]. Inhibiting autophagy caused insulin resistance [58], however, the pathophysiological role of changes in autophagy during insulin resistance on a cellular and systemic level remains controversial [59, 60]. Therefore, autophagy marker expression was analyzed basally and after insulin stimulation (2 nmol/L) in mature 3T3‐L1 adipocytes in conditions of regular glucose concentration in the cell culture media (1.0 g/L, 100 mg/dL, 5.6 mmol/L) versus high glucose concentration in the media (4.5 g/L, 450 mg/dL, 25 mmol/L).

Under regular glucose conditions, a stepwise increase in LAMP2 and MAP3LC3B expression was observed 90–360 min after insulin stimulation (Figure 2b,e). SIRT1 exhibited a differential expression pattern in response to insulin treatment under these conditions (Figure 2f). Under high glucose conditions, increased LAMP2 expression in response to insulin treatment was still detectable 360 min after treatment, however, MAP3LC3B expression levels remained unchanged (Figure 3b,e). SIRT1 expression levels exhibited a stepwise reduction in response to insulin treatment from 90 to 360 min after insulin treatment under these conditions (Figure 3f). LAMP1, BECLIN1, ATG5 and SIRT6 remained unchanged in both experimental settings. Future studies using targeted knock‐out approaches under regular and high glucose conditions as well as an analysis of insulin‐induced cell signaling events are needed to dissect the role of individual autophagy markers for adipocyte cellular insulin resistance.

Metformin is a pharmacologic agent used for decades as a first‐line treatment for type 2 diabetes mellitus in clinical medicine. It is known to improve systemic insulin resistance by its insulin‐sensitizing effect in patients with insulin resistance; however, the molecular mechanisms remain controversial [41]. During adipogenesis, metformin has been shown to impact on adipocyte differentiation through an AMPK‐Erk‐Akt dependent mechanism, while in mature adipocytes, metformin impacts on glucose metabolism through an AMPK‐PTEN dependent mechanism [61, 62, 63]. Furthermore, metformin blunts adipocyte pro‐inflammatory responses to LPS treatment [64]. Interestingly, Metformin‐induced AMPK activation promotes BECLIN1 dependent autophagy in gastric cancer cells [65], and restores autophagic flux in endothelial cells [66]. In the current study, metformin treatment induced BECLIN1 and ATG5 expression in mature 3T3‐L1 adipocytes in vitro (Figure 4). Since both BECLIN1 and ATG5 enhance autophagy [3, 21, 22, 23], these data indicate that metformin potentially enhances autophagy in adipocytes as well. Since inhibition of autophagy increases pro‐inflammatory cytokine secretion in adipose tissue [9], a hallmark of metabolically‐induced inflammation (metaflammation) in insulin resistance and type 2 diabetes, metformin might thus have a direct beneficial impact on adipocytes during metaflammation.

Although the experimental design presented in the current study was developed with caution, the following limitations apply: In studies on different human adipose tissues, bulk adipose tissue was employed; thus, the contribution of individual cell types to the overall results cannot be assessed. Since cellular composition of adipose tissues varies in obesity as opposed to normal weight, single cell transcriptomic data should address this shortfall in the future [67, 68, 69]. In studies on human adipose tissues and serum samples, comparison patients with normal weight were retrieved from commercially available mRNA banks, as described in the Methods section. Thus, although all precautions were taken to ensure the closest possible matching of patient characteristics, we cannot exclude that unwanted differences might bias the results. Also, it has been remarked that the expression levels of autophagy markers do not necessarily reflect autophagic flux as a marker of autophagic activity [48]. Consequently, data on the role of autophagy in the literature are conflicting [1, 60]. Future studies should implement targeted knock‐out approaches to further dissect the role of individual autophagy markers during adipocyte differentiation, in adipocyte physiology and in insulin resistance. Finally, future detailed analyses of cellular signaling events in response to insulin treatment in vitro are needed to dissect the role of individual autophagy markers in adipocyte cellular insulin resistance.

In summary, the current study provides a targeted expression analysis of an autophagy marker panel in patients with obesity versus normal weight. A differential expression pattern was observed. During 3T3‐L1 adipocyte differentiation in vitro, autophagy genes were induced at different time points, and autophagy markers exhibited a differential expression pattern in response to insulin treatment in adipocytes under regular glucose concentrations as compared to high glucose concentrations. Metformin induced BECLIN1 and ATG5 expression in mature adipocytes, potentially enhancing autophagy as a beneficial molecular effect in adipocytes during metaflammation.

## Author Contributions

**Edita Islami:** conceptualization, methodology, validation, formal analysis, investigation, data curation, writing – original draft, writing – review and editing, visualization. **Andreas Schmid:** conceptualization, methodology, validation, formal analysis, investigation, resources, data curation, writing – original draft, writing – review and editing, visualization. **Daniel Steger:** investigation. **Sebastian Köhler:** investigation. **Andreas Schäffler:** writing – review and editing, supervision, project administration. **Thomas Karrasch:** conceptualization, methodology, formal analysis, resources, data curation, writing – original draft, writing – review and editing, visualization, supervision, project administration, funding acquisition.

## Funding

This study was funded by a grant of the German Research Foundation (DFG) to T. Karrasch (KA 1846/4‐2).

## Conflicts of Interest

The authors declare no conflicts of interest.

## Supporting information


Supporting Information S1

## Data Availability

The data presented in this study are available on reasonable request from the corresponding author.
