# Supplementary material for: Obesity‐ and Glucose‐Dependent Differential Autophagy Marker Expression in Adipose Tissues and Adipocytes
Source: Obes Sci Pract. 2026 May 23;12(3):e70150. doi: 10.1002/osp4.70150 (PMC13240358; doi:10.1002/osp4.70150)
Supplement: Supplementary file 1 — Supporting Information S1 [file OSP4-12-e70150-s001.docx]

**Supplemental Table S1: Overview of baseline demographics in patients with and without obesity included in the analyses (mean [Range]).** Group differences were analysed by *Mann-Whitney* test (age, BMI) or *Pearson’s chi-squared* test (sex, diabetes), respectively. *P* values are shown.

|  | **lean (n=10)** | **obese (n= 40)** | ***P*** |
| --- | --- | --- | --- |
| **age [years]** | 43.9 ± 12.6 [26 – 71] | 41.2 ± 10.5 [24 – 69] | 0.658 |
| **sex [male / female]** | 2 / 8 | 8 / 32 | 1.000 |
| **BMI [kg/m^2^]** | 22.7 ± 1.7 [19.6 – 24.4] | 54.8 ± 6.7 [43.0 – 72.2] | **< 0.001** |
| **diabetes [yes / no]** | 0 / 10 | 17 / 23 | **0.011** |

**Supplemental Table S2: Primer sequences of target genes used in real-time RT-PCR analyses.**

| **target gene** | **forward primer** | **reverse primer** |
| --- | --- | --- |
|  | | |
| **human LAMP1** | 5’-ATGTGTTAGTGGCACCCAGG-3’ | 5’-TGTTCACAGCGTGTCTCTCC-3’ |
| **human LAMP2** | 5’-GCACAGTGAGCACAAATGAGTT-3’ | 5’-CCATGGTAGCCAGCAGACAA-3’ |
| **human BECN1** | 5’-GGGCTCCCGAGGGATGG-3’ | 5’-AGTTCCTGGATGGTGACACG-3’ |
| **human ATG5** | 5’-AAAGATGTGCTTCGAGATGTGT-3’ | 5’-CACTTTGTCAGTTACCAACGTCA-3’ |
| **human MAP1LC3B** | 5’-CCAACCAAAATCCCGGTGATAA-3’ | 5’-ATGCTGTGTCCGTTCACCAA-3’ |
| **human SIRT1** | 5’-GGGCTGCGGTTCCTACTG-3’ | 5’-CCGAACAGAAGGTTATCTGGCT-3’ |
| **human SIRT6** | 5’-CCCACGGAGTCTGGACCAT-3’ | 5’-CTCTGCCAGTTTGTCCCTG-3’ |
| **human GAPDH** | 5’-GAGTCCACTGGCGTCTTCAC-3’ | 5’-CCAGGGGTGCTAAGCAGTT-3’ |
|  | | |
| **murine LAMP1** | 5’-GCCCTGGAATTGCAGTTTGG-3’ | 5’-TGCTGAATGTGGGCACTAGG-3’ |
| **murine LAMP2** | 5’-CTTAGCTTCTGGGATGCCCC-3’ | 5’-GCACTGCAGTCTTGAGCTGT-3’ |
| **murine BECN1** | 5’-AGGAACTCACAGGAGCCATT-3’ | 5’-CTCCCCGATCAGAGTGAAGC-3’ |
| **murine ATG5** | 5’-ACACCCCTGAAATGAGTTTTCC-3’ | 5’-CATCCAGAGCTGCTTGTGGT-3’ |
| **murine MAP1LC3B** | 5’-GAGGGGACCCTAACCCCATA-3’ | 5’-TCGCTCTATAATCACCCGCC-3’ |
| **murine SIRT1** | 5’-TCGGCTACCGAGGTCCATA-3’ | 5’-ACAATCTGCCACAGCGTCAT-3’ |
| **murine SIRT6** | 5’-TGCAACCCACAAAACATGACC-3’ | 5’-GGAATCTCCAGCCCCAGATG-3’ |
| **murine GAPDH** | 5’-TGTCCGTCGTGGATCTGAC-3’ | 5’-AGGGAGATGCTCAGTGTTGG-3’ |

**Supplemental Table S3: Mean [Range] of autophagy marker expression in relation to GAPDH in scAT in patients with and without obesity.** Group differences were analysed by *Mann-Whitney* test and corrected for multiple testing using Bonferroni correction, and adjusted *P* values are shown.

|  | **lean (n=10) scAT** | **obese (n= 40) scAT** | ***P*** |
| --- | --- | --- | --- |
| **LAMP1 / GAPDH** | 0.042 ± 0.023 [0.022 – 0.095] | 0.172 ± 0.133 [0.037 – 0.555] | **< 0.001** |
| **LAMP2 / GAPDH** | 0.270 ± 0.138 [0.198 – 0.651] | 0.104 ± 0.053 [0.037 – 0.215] | **< 0.001** |
| **BECN1 / GAPDH** | 0.039 ± 0.020 [0.019 – 0.081] | 0.028 ± 0.013 [0.009 – 0.061] | 0.441 |
| **ATG5 / GAPDH** | 0.223 ± 0.138 [0.121 – 0.576] | 0.050 ± 0.088 [0.008 – 0.479] | **< 0.001** |
| **MAP1LC3B / GAPDH** | 0.557 ± 0.347 [0.323 – 1.491] | 0.186 ± 0.089 [0.056 – 0.366] | **< 0.001** |
| **SIRT1 / GAPDH** | 0.0019 ± 0.0007 [0.0010 – 0.0032] | 0.0011 ± 0.0005 [0.0003 – 0.0025] | **0.005** |
| **SIRT6 / GAPDH** | 0.00014 ± 0.00011 [0.00004 – 0.00040] | 0.00085 ± 0.00088 [0.00001 – 0.00338] | **0.004** |

**Supplemental Table S4: Mean [Range] of autophagy marker expression in relation to GAPDH in visAT as compared to sc AT in patients with obesity.** Group differences were analysed by *Wilcoxon* test and corrected for multiple testing using Bonferroni correction, and adjusted *P* values are shown.

|  | **obese (n= 40) scAT** | **obese (n= 40) visAT** | ***P*** |
| --- | --- | --- | --- |
| **LAMP1 / GAPDH** | 0.172 ± 0.133 [0.037 – 0.555] | 0.167 ± 0.141 [0.019 – 0.678] | 1.000 |
| **LAMP2 / GAPDH** | 0.104 ± 0.053 [0.037 – 0.215] | 0.112 ± 0.070 [0.027 – 0.340] | 1.000 |
| **BECN1 / GAPDH** | 0.028 ± 0.013 [0.009 – 0.061] | 0.031 ± 0.020 [0.008 – 0.118] | 1.000 |
| **ATG5 / GAPDH** | 0.050 ± 0.088 [0.008 – 0.4799 | 0.034 ± 0.019 [0.009 – 0.085] | 0.616 |
| **MAP1LC3B / GAPDH** | 0.186 ± 0.089 [0.056 – 0.3669 | 0.206 ± 0.111 [0.043 – 0.497] | 1.000 |
| **SIRT1 / GAPDH** | 0.0011 ± 0.0005 [0.0003 – 0.0025] | 0.0008 ± 0.0005 [0.0001 – 0.0027] | **0.010** |
| **SIRT6 / GAPDH** | 0.00085 ± 0.00088 [0.00001 – 0.00338] | 0.00050 ± 0.00042 [0.00002 – 0.0017] | 0.487 |
